# Long-Term Body Mass Index Variability and Adverse Cardiovascular Outcomes

**DOI:** 10.1001/jamanetworkopen.2024.3062

**Published:** 2024-03-21

**Authors:** Zakaria Almuwaqqat, Qin Hui, Chang Liu, Jin J. Zhou, Benjamin F. Voight, Yuk-Lam Ho, Daniel C. Posner, Jason L. Vassy, J. Michael Gaziano, Kelly Cho, Peter W. F. Wilson, Yan V. Sun

**Affiliations:** 1 Veterans Affairs Atlanta Healthcare System, Decatur, Georgia; 2 Division of Cardiology, Department of Medicine, Emory University School of Medicine, Atlanta, Georgia; 3 Department of Epidemiology, Emory University Rollins School of Public Health, Atlanta, Georgia; 4 Department of Medicine and Biostatistics, University of California, Los Angeles; 5 Veterans Affairs Phoenix Healthcare System, Phoenix, Arizona; 6 Corporal Michael J. Crescenz VA Medical Center, Philadelphia, Pennsylvania; 7 Department of Systems Pharmacology and Translational Therapeutics, Department of Genetics, University of Pennsylvania, Philadelphia\; 8 Massachusetts Veterans Epidemiology Research and Information Center, VA Boston Healthcare System, Boston; 9 Department of Medicine, Brigham and Women’s Hospital, Harvard Medical School, Boston, Massachusetts; 10 Division of Aging, Department of Medicine, Brigham and Women’s Hospital, Harvard Medical School, Boston, Massachusetts

## Abstract

**Importance:**

Body mass index (BMI; calculated as weight in kilograms divided by height in meters squared) is a commonly used estimate of obesity, which is a complex trait affected by genetic and lifestyle factors. Marked weight gain and loss could be associated with adverse biological processes.

**Objective:**

To evaluate the association between BMI variability and incident cardiovascular disease (CVD) events in 2 distinct cohorts.

**Design, Setting, and Participants:**

This cohort study used data from the Million Veteran Program (MVP) between 2011 and 2018 and participants in the UK Biobank (UKB) enrolled between 2006 and 2010. Participants were followed up for a median of 3.8 (5th-95th percentile, 3.5) years. Participants with baseline CVD or cancer were excluded. Data were analyzed from September 2022 and September 2023.

**Exposure:**

BMI variability was calculated by the retrospective SD and coefficient of variation (CV) using multiple clinical BMI measurements up to the baseline.

**Main Outcomes and Measures:**

The main outcome was incident composite CVD events (incident nonfatal myocardial infarction, acute ischemic stroke, and cardiovascular death), assessed using Cox proportional hazards modeling after adjustment for CVD risk factors, including age, sex, mean BMI, systolic blood pressure, total cholesterol, high-density lipoprotein cholesterol, smoking status, diabetes status, and statin use. Secondary analysis assessed whether associations were dependent on the polygenic score of BMI.

**Results:**

Among 92 363 US veterans in the MVP cohort (81 675 [88%] male; mean [SD] age, 56.7 [14.1] years), there were 9695 Hispanic participants, 22 488 non-Hispanic Black participants, and 60 180 non-Hispanic White participants. A total of 4811 composite CVD events were observed from 2011 to 2018. The CV of BMI was associated with 16% higher risk for composite CVD across all groups (hazard ratio [HR], 1.16; 95% CI, 1.13-1.19). These associations were unchanged among subgroups and after adjustment for the polygenic score of BMI. The UKB cohort included 65 047 individuals (mean [SD] age, 57.30 (7.77) years; 38 065 [59%] female) and had 6934 composite CVD events. Each 1-SD increase in BMI variability in the UKB cohort was associated with 8% increased risk of cardiovascular death (HR, 1.08; 95% CI, 1.04-1.11).

**Conclusions and Relevance:**

This cohort study found that among US veterans, higher BMI variability was a significant risk marker associated with adverse cardiovascular events independent of mean BMI across major racial and ethnic groups. Results were consistent in the UKB for the cardiovascular death end point. Further studies should investigate the phenotype of high BMI variability.

## Key Points

**Question:**

Is increased BMI variability over time associated with adverse cardiovascular events independent of traditional risk factors and mean BMI, and is this associated with genetic variations?

**Findings:**

In this cohort study including 157 410 individuals from 2 large study cohorts, BMI variability over time was significantly associated with adverse cardiovascular events independent of the mean overall BMI and its genetic risk score.

**Meaning:**

These findings suggest that highly variable BMI is associated with adverse cardiovascular events and may explain some of the residual cardiovascular risk.

## Teaser

This cohort study evaluates the association between body mass index variability over time and incident cardiovascular disease events in 2 distinct cohorts.

## Introduction

Atherosclerotic cardiovascular disease (ASCVD) is a leading cause of morbidity and mortality globally.^1^ Obesity, defined as body mass index (BMI; calculated as weight in kilograms divided by height in meters squared) of 30 or greater, is associated with multiple metabolic derangements that promote inflammatory and atherogenic interior milieu, leading to cardiovascular morbidity and mortality.^2,3,4,5,6,7,8^ However, the prognostic value for increasing BMI changes remains controversial, depending on whether it is defined by higher longitudinal BMI variance or by relative or absolute BMI changes. Elevated BMI fluctuation may lead to excessive changes in blood pressure (BP), heart rate, and glycemic control that could impose stress on the cardiovascular system. Population-based studies have demonstrated that higher BMI fluctuation is associated with overall and cardiovascular-related mortality.^9,10^ Similar findings were reported in a National Health and Nutrition Examination Survey study.^11^ Additionally, BMI fluctuation has been reported as a significant risk factor associated with death and secondary coronary events in patients with coronary artery disease.^12^ However, other studies have demonstrated that sustained BMI changes are more strongly associated with outcomes than BMI fluctuation is.^13,14^ Most of these studies are limited by the lack of consideration of modern health care and temporal trends in cardiovascular mortality and morbidity rates. Moreover, to our knowledge, no prior studies have investigated this association in a racially and ethnically diverse sample while excluding potential confounding effects related to cancer.

We used electronic health record (EHR) data for racially and ethnically diverse participants from the Million Veteran Program (MVP) to investigate the association between incident adverse cardiovascular events and variabilities in clinically measured BMI through multiple health care visits. We hypothesized that higher BMI variability across multiple clinical visits would be associated with increased risk for adverse cardiovascular events. We also investigated whether the polygenic risk score of BMI moderates this association. We have also sought to investigate the same question in the UK Biobank (UKB) study as an independent replication cohort with distinct demographic composition.

## Methods

This cohort study was approved by the Department of Veterans Affairs (VA) central institutional review board, in accordance with the principles outlined in the Declaration of Helsinki. Informed consent was obtained from all participants. The study was conducted according to the Strengthening the Reporting of Observational Studies in Epidemiology (STROBE) reporting guideline.

### MVP Cohort

#### Sample Characteristics

The MVP cohort has been previously described.^15^ The MVP recruited participants at more than 60 VA medical centers across the United States, starting in 2011. Structured data from the VA EHR were used to derive the outcomes and phenotypic variables. The quality and reliability of VA EHR data has been previously demonstrated across a variety of contexts relevant to this study.^16,17,18^ We extracted EHRs using the national VA Corporate Data Warehouse.^19^ We also ascertained clinical outcomes from the VA records using data from the Centers for Medicaid & Medicare Services (CMS) and National Death Index (NDI). Participants with cancer were excluded to control for potential confounding bias due to cancer progression or treatment.

#### Main Exposure

We assessed variabilities in clinically measured BMI through multiple health care visits up to the enrollment date. Our main exposure was the coefficient of variation (CV) for BMI based on the SD in BMI change over multiple visits divided by the mean BMI across these visits.^20^ We adjusted for the mean BMI in all analyses.^20^

#### Other Risk Factors

Patient characteristics were assessed at baseline, defined as an outpatient VA clinic visit close to MVP enrollment date.^21^ Participants were categorized into harmonized ancestry and race and ethnicity groups, including Hispanic, non-Hispanic Black, and non-Hispanic White.^22^ The harmonized ancestry and race and ethnicity method uses genetically inferred ancestry to refine self-identified ethnicity in 3 ways: identify individuals whose self-identified ethnicity is likely inaccurate, reconcile conflicts among multiple self-identified ethnicity sources, and impute missing ethnic information when the predictive confidence is high. Whether to include race and ethnicity in clinical prognostic models can be controversial but remains the standard of care in CVD risk estimation and prevention.^23^ A risk factor was considered present if 2 or more codes for a given risk factor were identified. In our analysis, we adjusted for hypertension (yes vs no), diabetes (yes vs no), smoking history (current use vs not), high-density lipoprotein cholesterol (HDL) level (continuous), systolic BP (continuous), BP therapy (yes vs no), total cholesterol level (continuous), and statin use (current use vs not). Physical activity was self-reported using the MVP lifestyle survey. Baseline diabetes status was defined as any prescription for diabetes medication prior to enrollment plus 1 *International Classification of Diseases, Ninth Revision *(*ICD-9*) 250.xx (or equivalent *International Statistical Classification of Diseases and Related Health Problems, Tenth Revision *[*ICD-10*]) code in combination with a VA primary care visit or at least 2 total *ICD-9* or *ICD-10* codes. Diagnoses and tobacco use were identified using diagnosis codes available between June 1991 and August 2018. Statin therapy and antihypertensive therapy were defined as an active prescription for a relevant medication on the enrollment date. Medications and *ICD-9* and *ICD-10* codes used for inclusion and exclusion are available elsewhere.^24^

#### Polygenic Risk Scores

Genetic variants in MVP were genotyped with an Affymetrix microarray (Thermo Fisher Scientific).^25^ For variants not genotyped in MVP, dosages were imputed using the 1000 Genomes Project reference panel^26^ and African Genome Resources panel.^27^ The human genome reference assembly version GRCh37/hg19 was used to map genetic variants. An algorithm developed to harmonize genetic ancestry and self-reported race and ethnicity was used to estimate and define individual race and ethnicity as either European, African, or Hispanic. Single nucleotide polymorphisms with imputation quality greater than 0.5, Hardy-Weinberg Equilibrium *P* value greater than 10^−20^, missing rate less than 0.05, and minor allele frequency greater than 0.01 were included in the analysis.^22^ A genomewide association study was conducted for BMI. Autosomal single nucleotide polymorphisms were analyzed using logistic regression models implemented in PLINK version 2.0.^28^ Under the assumption of additive genetic mode, linear regression models were adjusted for age, sex, and top 10 principal components derived from the full genomics data after Linkage Disequilibrium parameters.^29^ The polygenic risk score of BMI was derived previously and comprised of 2.1 million common variants and tested in more than 300 000 individuals.^30^

#### Outcomes and Follow-Up

The primary end point was a composite outcome of incident arterial ischemic stroke (AIS), myocardial infarction (MI), and cardiovascular death, derived from relevant *ICD-9* and *ICD-10* revision codes and Current Procedural Terminology codes occurring in the EHR between June 1991 and October 2018. Cardiovascular death was defined as having any of the following *International Statistical Classification of Diseases, Tenth Revision, Clinical Modification *(*ICD-10-CM*) diagnosis codes as the primary cause of death in NDI: I10, I11, I13, I16, I20 to I25, I46, I63, I67, I70, I74, I75, and G45. *ICD-9*, *ICD-10*, or CMS codes from validated VA algorithms were used to define incident AIS and MI events.^31,32^ Follow-up time for each CVD outcome began at MVP enrollment date and ended at date of first outcome, death, or administrative censoring. Participants who had a nonfatal CVD event were included in the risk set for other subsequent CVD events.^33^

### UKB Cohort

#### Sample Characteristics

We analyzed data from 65 047 participants with at least 3 longitudinal BMI measurements from primary care records within a time window of more than 2 years before and at enrollment and who did not have cancer, coronary artery disease, or stroke at the enrollment visit in the UKB.^34^ Participants aged 37 to 73 years were enrolled from the general population of the United Kingdom between 2006 and 2010. At recruitment, data were collected using a standardized questionnaire on sociodemographic characteristics, health status and physician-diagnosed medical conditions, family history, and lifestyle factors, including smoking history and frequency of alcohol intake. Physical and functional measurements were obtained, including height, weight, BMI, and BP measures. We also used the Hospital Episode Statistics data, linked to UKB, which cover all hospital admissions up until 2023, dating back to 1997 for England, 1998 for Wales, and 1981 for Scotland. Hospital Episode Statistics uses *ICD-9* or *ICD-10* codes to record diagnosis information and Office of Population, Censuses and Surveys: Classification of Interventions and Procedures, version 3 or 4 to code operative procedures.

#### Main Exposure

We used clinical values for BMI that were measured during multiple health care visits from the UKB assessment center. We then calculated the CV for BMI as the SD in BMI change over multiple visits divided by the mean BMI across these visits. A high CV indicated a relatively higher SD compared with the mean BMI, which indicated a greater variability in BMI.

#### Other Risk Factors

As explained elsewhere,^35^ demographics and relevant risk factor variables were obtained at enrollment, including age, sex, BMI, hypertension, smoking, diabetes, HDL, total cholesterol level, estimated glomerular filtration rate (eGFR) (calculated as eGFR = 175 × serum creatinine^−1.154^ × age^−0.203^ × 1.212 (if patient is Black) × 0.742 (if patient is female), and summed metabolic equivalent task minutes per week for all physical activity.^36^ In the UKB, race and ethnicity were self-reported and categorized as Black, Chinese, multiple races or ethnicities, South Asian, White, and other (ie, any other race or ethnicity not already specified); however, we only included White participants in this analysis.

#### Outcomes

The primary end point was a composite outcome of incident ischemic stroke, incident MI, and cardiovascular death. Using Hospital Episode Statistics data,^37^ definitions for clinical end points were generated using the first occurrence of *ICD-10* code within primary care data, hospital inpatient data, death register records, and self-reported medical condition codes reported at the baseline or subsequent UKB assessment center visit.

### Statistical Analysis

In the MVP and UKB separately, the Fine and Gray competing risk models^38^ were used to estimate hazard ratios (HRs) and 95% CIs for the association between BMI variability as measured by the CV and incident CVD events. The proportional hazards assumption was checked using the Schoenfeld residuals.

In the MVP, separate models were conducted for composite CVD and each component outcome of CVD events (AIS, MI, or cardiovascular death), stratified by harmonized genetic ancestry and race and ethnicity group. First, we examined the unadjusted association of BMI variability. Then, we performed sequential adjustment using multivariate hazard regression models. Model 1 adjusted for potential confounding factors, including age, sex, mean BMI, diabetes, smoking history, HDL, systolic BP, BP therapy, total cholesterol level, and statin use. Model 2 adjusted for the BMI polygenic risk score (PRS) in addition to covariates from model 1. Finally, model 3 included model 1 covariates after adjustment for physical activity. We then examined whether our results were only significant in a certain subgroup by age (≥65 vs <65 years), sex, prevalent diabetes, and ranges of mean BMI.

In the UKB, we performed sequential adjustment for BMI variability using multivariate hazard regression models. Model 1 adjusted for potential confounding factors, including age, sex, mean BMI, diabetes, smoking history, HDL, systolic BP, BP therapy, total cholesterol level, eGFR, and statin use. Models 2 and 3 were further adjusted for the BMI PRS and physical activities, respectively. All analyses were conducted using R statistical software version 4.0.2 (R Project for Statistical Computing). Statistical significance was defined as a 2-sided *P* < .05. Data were analyzed from September 2022 to September 2023.

## Results

**Figure 1.**
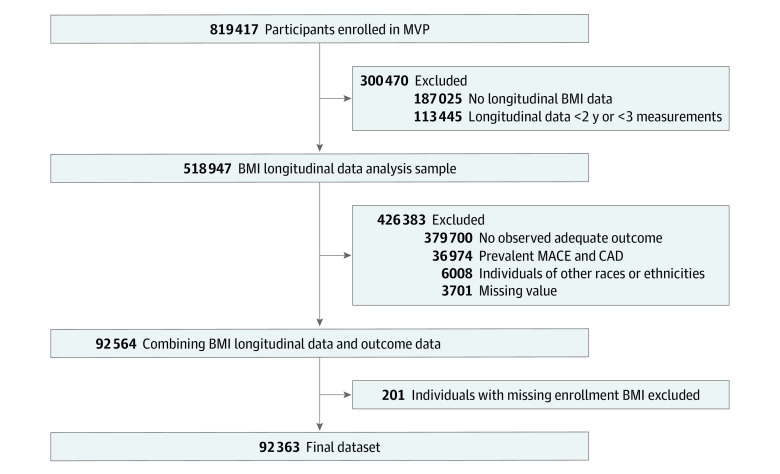
Participant Inclusion Flowchart

The MVP study sample comprised 92 363 participants, including 9695 Hispanic participants, 22 488 non-Hispanic Black participants, and 60 180 non-Hispanic White participants (Figure 1). A total of 81 675 participants (88%) were men (Table 1). The overall mean (SD) age was 56.7 (14.1) years, but 7 years younger for Hispanic participants (49.4 [15.6] years) and 3 years younger for non-Hispanic Black participants (53.2 [12.6] years). At baseline, 31 688 participants (34%) had active prescriptions for stains and 42 740 participants (46%) had active prescriptions for antihypertensive therapies. At baseline, the mean (SD) BMI was 30.36 (5.98), and BMI measurements were obtained over a median (IQR) of 10.4 (6.3-13.2) years. As illustrated in eTable 1 in Supplement 1, the UKB sample included 65 047 individuals (mean [SD] age, 57.30 (7.77) years; 38 065 [59%] female) and only included White participants. Additionally, this sample was leaner, with mean (SD) BMI of 26.83 (4.60).

**Table 1.** Baseline Characteristics of the Million Veteran Project Sample According to Harmonized Ancestry and Race and Ethnicity

Characteristic	Participants, No. (%)			
	Total cohort (N = 92 363)	Harmonized ancestry and race and ethnicity		
		Hispanic (n = 9695)	Non-Hispanic Black (n = 22 488)	Non-Hispanic White (n = 60 180)
Sex				
Female	10 688 (12)	1092 (11)	3422 (15)	6174 (10)
Male	81 675 (88)	8603 (89)	19 066 (85)	54 006 (90)
Age, mean (SD), y	56.7 (14.1)	50.8 (14.6)	53.9 (11.7)	58.7 (14.4)
HDL, mean (SD), mg/dL	4.87 (1.64)	45.8 (1.51)	52.3 (1.73)	47.0 (1.60)
SBP, mean (SD), mm Hg	130.9 (14.1)	129.2 (13.8)	132.4 (14.6)	130.7 (13.9)
Treatment for BP	42 740 (46)	3776 (39)	10 881 (48)	28 083 (47)
Diabetes	21 795 (24)	2410 (25)	6425 (29)	12 960 (22)
Smoking	8852 (10)	1050 (11)	2242 (10)	5560 (9)
Statin use	31 688 (34)	2758 (28)	6785 (30)	22 145 (37)
BMI at enrollment	30.36 (5.98)	31.01 (5.83)	30.92 (6.12)	30.05 (5.92)
No. of BMI measurements	24 (20)	23 (21)	26 (22)	23 (20)
Longitudinal BMI, mean (SD)	30.39 (5.64)	30.85 (5.47)	30.69 (5.77)	30.21 (5.61)
SD of longitudinal BMI, mean (SD)	1.42 (0.82)	1.45 (0.84)	1.47 (0.81)	1.40 (0.82)
CV of longitudinal BMI, mean (SD)	0.0466 (0.0247)	0.0468 (0.025)	0.0479 (0.025)	0.0461 (0.025)
Follow-up time, mean (SD), y	3.7 (2.0)	3.5 (2.0)	3.7 (1.9)	3.7 (2.0)
Outcome, No. (%) [incidence per 100 PY]				
Acute ischemic stroke	1329 (3) [0.39]	99 (1) [0.29]	397 (2) [0.48]	833 (1) [0.37]
Myocardial infarction	3044 (1) [0.90]	218 (2) [0.65]	667 (3) [0.80]	2159 (4) [0.97]
Cardiovascular death	810 (1) [0.24]	52 (1) [0.15]	153 (1) [0.18]	605 (1) [0.27]
MACE	4811 (5) [1.44]	345 (4) [1.03]	1110 (5) [1.35]	3356 (6) [1.53]

Abbreviations: BMI, body mass index (calculated as weight in kilograms divided by height in meters squared); CV, coefficient of variation; HDL, high-density lipoprotein; MACE, major adverse cardiac events; SBP, systolic blood pressure.

SI conversion factor: To convert HDL to millimoles per liter, multiply by 0.0259.

During a median follow-up of 3.8 (5th-95th percentile, 3.5) years, 4811 participants (5%) experienced at least 1 CVD event, among whom 3044 (3%) experienced an MI, 1329 (1%) experienced an AIS, and 810 (1%) died of a CVD event, including multiple possible events for each participant, for a total of 4811 CVD events in the MVP cohort and 6934 CVD events in the UKB cohort. The crude incidence rate of composite CVD events was greatest among non-Hispanic White participants, at 1.53 per 100 person-years (PY), slightly lower for non-Hispanic Black participants (1.35 events per 100 PY), and much lower for Hispanic participants (1.03 events per 100 PY).

### Primary Analyses: MVP Cohort

#### BMI Variability and Composite CVD Events

Among non-Hispanic Black participants, each 1-SD of BMI CV, as a measure of BMI variability, was associated with an 8% higher risk for adverse events (HR, 1.08; 95% CI, 1.00-1.16). However, among participants from both Hispanic and non-Hispanic White ancestries, BMI CV was not significantly associated with adverse event risk (Hispanic: HR, 1.12; 95% CI, 0.98-1.28; non-Hispanic White: HR, 0.99; 95% CI, 0.94-1.04), using the same univariate model (eTable 2 in Supplement 1). In contrast, after adjustment for covariates, including age, sex, mean BMI, diabetes, smoking, systolic BP, antihypertensive medications, and cholesterol and HDL levels, BMI variability was significantly associated with composite adverse events among Hispanic participants (HR, 1.24; 95% CI, 1.14-1.35), non-Hispanic Black participants (HR, 1.16; 95% CI, 1.10-1.21), and non-Hispanic White participants (HR, 1.15; 95% CI, 1.12-1.19). Finally, the CV of BMI was associated with 16% higher risk for composite CVD events across all groups (HR, 1.16; 95% CI, 1.13-1.19). Further adjustment for BMI PRS or physical activity did not impact this association (Table 2). Results were similar in analysis conducted in the overall cohort.

**Table 2.** Associations Between Body Mass Index Variability and Composite Adverse Cardiovascular Events

Harmonized ancestry and race and ethnicity	HR (95%CI)^a^	*P* value
Hispanic		
Model 1^b^	1.24 (1.14-1.35)	<.001
Model 2^c^	1.24 (1.14-1.35)	<.001
Model 3^d^	1.25 (1.10-1.40)	.003
Non-Hispanic Black		
Model 1^b^	1.16 (1.10-1.21)	.001
Model 2^c^	1.16 (1.10-1.21)	<.001
Model 3^d^	1.13 (1.05-1.22)	.005
Non-Hispanic White		
Model 1^b^	1.15 (1.12-1.19)	<.001
Model 2^c^	1.15 (1.12-1.19)	<.001
Model 3^d^	1.11 (1.07-1.16)	<.001
Overall cohort		
Model 1^b^	1.16 (1.13-1.19)	<.001
Model 2^c^	1.16 (1.13-1.19)	<.001
Model 3^d^	1.13 (1.09-1.16)	<.001

Abbreviation: HR, hazard ratio.

^a^ Per 1 SD of body mass index variability.

^b^ Model 1 adjusted for age, sex, mean body mass index, diabetes, smoking history, high-density lipoprotein, systolic blood pressure, blood pressure therapy, total cholesterol level, and statin use.

^c^ Model 2 adjusted for model 1 covariates plus polygenic risk score for body mass index trait.

^d^ Model 3 adjusted for model 1 covariates plus physical activity (n = 45 038).

#### BMI Variability and Specific CVD Events

Summarized in Table 3, elevated BMI CV was associated with higher risk for incident MI among Hispanic participants (HR, 1.18; 95% CI, 1.05-1.31), non-Hispanic Black participants (HR, 1.12; 95% CI, 1.05-1.19) and non-Hispanic White participants (HR, 1.11; 95% CI, 1.07-1.16). Similarly, each 1-SD increment of BMI CV was associated with incident AIS among Hispanic participants (HR, 1.31; 95% CI, 1.08-1.46) and non-Hispanic Black participants (HR, 1.16; 95% CI, 1.07-1.25). In contrast, there was no significant association among non-Hispanic White participants (HR, 1.04; 95% CI, 0.97-1.12). Finally, the strongest association was observed with cardiovascular death: each 1-SD increment of BMI CV was associated with 53% increased risk among Hispanic participants (HR, 1.53; 95% CI, 1.30-1.76), 27% increased risk among non-Hispanic Black participants (HR, 1.27; 95% CI, 1.13-1.41), and 41% increased risk among non-Hispanic White participants (HR, 1.41; 95% CI, 1.34-1.48).

**Table 3.** Associations Between Body Mass Index Variability and Specific Adverse Cardiovascular Events

Outcome	HR (95%CI)^a^	*P* value
Myocardial infarction		
Hispanic	1.18 (1.05-1.31)	.02
Non-Hispanic Black	1.12 (1.05-1.19)	.003
Non-Hispanic White	1.11 (1.07-1.16)	.009
Overall cohort	1.12 (1.08-1.16)	<.001
Acute ischemic stroke		
Hispanic	1.31 (1.08-1.46)	.02
Non-Hispanic Black	1.16 (1.07-1.25)	.002
Non-Hispanic White	1.04 (0.97-1.12)	.28
Overall cohort	1.10 (1.04-1.16)	<.001
Cardiovascular death		
Hispanic	1.53 (1.30-1.76)	<.001
Non-Hispanic Black	1.27 (1.13-1.41)	.006
Non-Hispanic White	1.41 (1.34-1.48)	<.001
Overall cohort	1.38 (1.32-1.44)	<.001

Abbreviation: HR, hazard ratio.

^a^ Per 1 SD of body mass index variability. Model adjusted for age, sex, mean body mass index, diabetes, smoking history, high-density lipoprotein, systolic blood pressure, blood pressure therapy, total cholesterol level, and statin use.

### Secondary Analysis

**Figure 2.**
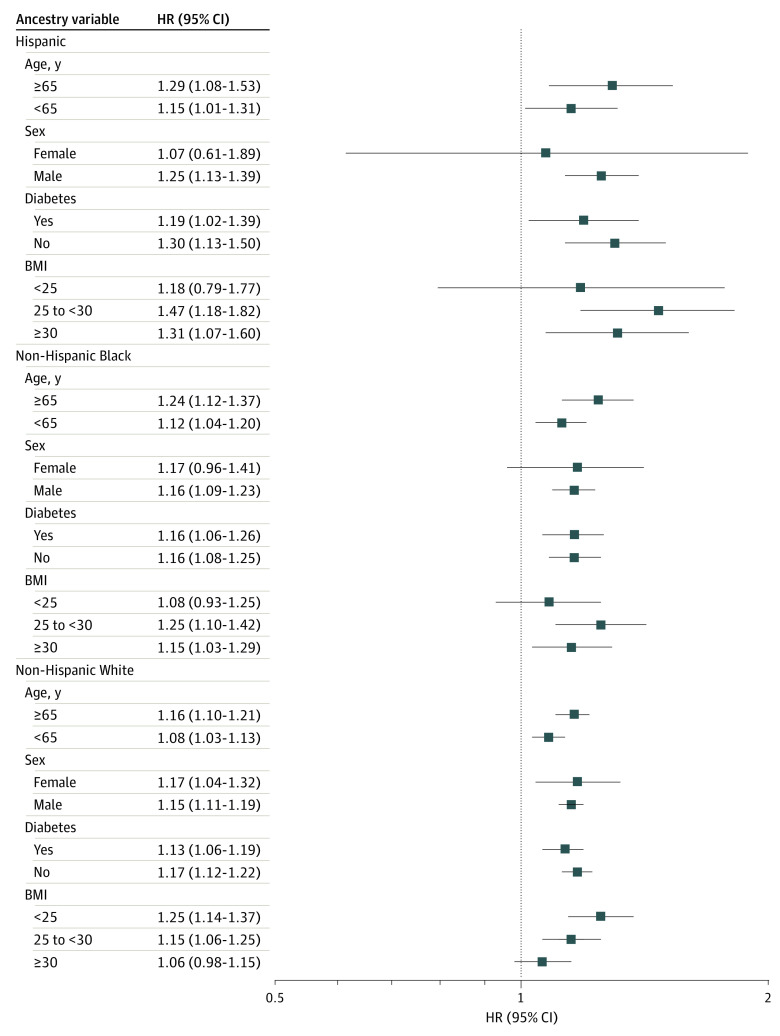
Subgroup Analysis of the Associations Between Body Mass Index (BMI) Variability and Adverse Cardiovascular Events by Different Groups BMI is calculated as weight in kilograms divided by height in meters squared. HR indicates hazard ratio.

In subgroup analyses, most associations between BMI variability and adverse CVD events were consistent across risk factor subgroups (Figure 2). Among Hispanic and non-Hispanic White participants, there were significant interactions between BMI variation and baseline BMI, in which the association was more robust among those with normal weight or overweight (Figure 2). Younger participants had more pronounced associations, but this was only significant in the non-Hispanic Black group (Figure 2). Female participants had a less pronounced association, particularly non-Hispanic White participants (Figure 2). Finally, the association was stronger among Hispanic participants (Figure 2). After considering multiple testing of 4 subgroup analyses, the results were not statistically significant for most interactions.

### Replication in the UKB Cohort

To validate findings from the MVP, we assessed a comparable sample from the UKB free of ASCVD and cancer at enrollment. Participants with 1-SD higher BMI CV had a 8% higher risk of cardiovascular death (HR, 1.08; 95% CI, 1.04-1.11) but no significant difference in the risk for the composite outcome of incident coronary artery disease, stroke, and cardiovascular death (HR, 1.02; 95% CI, 0.995-1.04) (eTable 3 in Supplement 1). Results were similar when analyzed the findings by sex (eTable 4 in Supplement 1)

## Discussion

In this large racially and ethnically diverse cohort study of veterans using EHR-derived data from the MVP study, we found that BMI variability, as measured by CV of longitudinal BMI, was an independent risk factor for adverse cardiovascular events, conditional on traditional ASCVD risk factors, mean BMI, and PRS of BMI. Furthermore, we found that the association between BMI variability and stroke was stronger in non-Hispanic Black participants, whereas the association between BMI variability and cardiovascular death was higher in non-Hispanic White participants. The same association between BMI variability and cardiovascular death was consistent, albeit with smaller association, in an independent cohort of men and women from a different ancestral background and geographic region (UKB). These findings suggest that highly changing BMI may represent a high-risk phenotype that needs further exploration and characterization.

The association between BMI variation and incident cardiovascular events is inconsistent across previous studies. A 1989 analysis comparing the risk of coronary heart disease among more than 2000 men found that individuals who reported gain and loss of weight had the highest incidence for coronary heart disease compared with those who reported little or no change in weight.^39^ Similarly, a 1991 analysis from the Framingham Heart Study found that greater body weight variability was associated with adverse cardiovascular events among individuals without CVD.^40^ Some reports have focused on secondary CVD events among individuals with coronary artery disease and those with diabetes.^12,41^ However, these studies examined mainly White men, with no replication of findings among Black or other individuals. Moreover, other studies have failed to firmly establish the association between BMI variability and adverse events.^13,14,42^ It is possible that the inconsistent findings could be related to the lack of diverse participants, inclusion of participants with cancer, or differential associations across certain groups of individuals. Our study used EHR-based large biobank cohorts to provide robust evidence of the association between BMI variability and adverse CVD events in multiple racial and ethnic groups with consideration of potential genetic influence.

Mechanisms underlying the association between BMI variability and adverse cardiovascular events remain unclear. Several hypotheses have been postulated to explain this association. Animal studies have shown that weight cycling, as defined by weight loss followed by weight gain, is associated with loss of energy hemostasis and a rise in hunger and fall in satiety hormones, leading to adipocyte hyperplasia and maladaptive excess visceral fat accumulation.^43,44^ Human studies have linked weight change to vascular function deterioration and elevated BP via visceral fat accumultion.^45^ Epidemiological studies have consistently shown that weight change is likely to increase body fat and is associated with unfavorable metabolic and psychosocial attributes.^46,47,48^ These findings were interpreted as a result of potential overshoot in correcting cardiac risk markers, including hypertension, heart rate, cholesterol, and visceral adiposity, consistent with the repeated overshoot theory promoting an unnecessary load on the cardiovascular system.^49,50^ It is possible that weight or BMI change is a correlate for dietary and psychosocial factors that predispose the individual to CVD events. However, it is important to recognize highly variable BMI as a high-risk phenotype independent of the known risk pathways involving the association between obesity and CVD, as evident by the lack of association with BMI PRS. Results from our study highlight the need for future research focusing on understanding metabolic derangements associated with BMI variability and adverse cardiovascular events. The growing resources in large cohorts, such as the MVP and UKB, enable robust association analyses of genetic and molecular factors, as well as lifestyle factors to uncover underlying pathophysiological mechanisms.

### Limitations

Limitations of this study include the overall relative underrepresentation of female participants in the MVP cohort. However, we included more than 10 000 female participants in this analysis. Additionally, our results were validated in the UKB, which has a more balanced sex distribution. We also found that the association of variable BMI to be the strongest with cardiovascular mortality and less evident for MI, which is possibly due to underascertainment of MI in the MVP population.^21^ Possible explanations for the differences in associations between the MVP and UKB cohorts could be related to the significantly greater number of BMI measurement in the MVP study compared with the UKB. Other strengths include the replication of results in a second independent cohort from the UK. These findings confirm the consistency and generalizability of our findings.

## Conclusions

In this large cohort study of racially and ethnically diverse veterans using EHR-derived data from the MVP, we found that greater BMI fluctuation across multiple clinical visits was an independent risk factor associated with adverse CVD events irrespective of traditional CVD risk factors and genetic risk score for BMI. These findings suggest that greater BMI changes could be related to a high-risk phenotype for CVD, with unique metabolic and genetic mechanisms. Future studies should focus on identifying molecular risk pathways mediating this association.

## Notes

Supplement 1. **eTable 1.** UK Biobank Cohort Characteristics **eTable 2.** Unadjusted Associations Between Body Mass Index Variability and Adverse Cardiovascular Events **eTable 3.** Associations Between Body Mass Index Variability and Adverse Cardiovascular Events From the UK Biobank **eTable 4.** Sex-Specific Associations Between Body Mass Index Variability and Adverse Cardiovascular Events From the UK Biobank **eAppendix.** VA Million Veteran Program: Core Acknowledgment for Publications

Supplement 2.
Data Sharing Statement
