# Supplementary material for: Long-Term Body Mass Index Variability and Adverse Cardiovascular Outcomes
Source: JAMA Netw Open. 2024 Mar 21;7(3):e243062. doi: 10.1001/jamanetworkopen.2024.3062 (PMC10958234; doi:10.1001/jamanetworkopen.2024.3062)
Supplement: Supplement 1. — eTable 1. UK Biobank Cohort Characteristics eTable 2. Unadjusted Associations Between Body Mass Index Variability and Adverse Cardiovascular Events eTable 3. Associations Between Body Mass Index Variability and Adverse Cardiovascular Events From the UK Biobank eTable 4. Sex-Specific Associations Between Body Mass Index Variability and Adverse Cardiovascular Events From the UK Biobank eAppendix. VA Million Veteran Program: Core Acknowledgment for Publications [file jamanetwopen-e243062-s001.pdf]

Supplemental Online Content

Almuwaqqat Z, Hui Q, Liu C, et al. Long-term body mass index variability and adverse cardiovascular outcomes. *JAMA Netw Open*. 2024;7(3):e243062. doi:10.1001/jamanetworkopen.2024.3062

eTable 1. UK Biobank Cohort Characteristics
eTable 2. Unadjusted Associations Between Body Mass Index Variability and Adverse Cardiovascular Events
eTable 3. Associations Between Body Mass Index Variability and Adverse Cardiovascular Events From the UK Biobank
eTable 4. Sex-Specific Associations Between Body Mass Index Variability and Adverse Cardiovascular Events From the UK Biobank
eAppendix. VA Million Veteran Program: Core Acknowledgment for Publications

This supplemental material has been provided by the authors to give readers additional information about their work.

© 2024 Almuwaqqat Z et al. *JAMA Network Open.*

**Supplemental eTable 1. UK Biobank Cohort Characteristics White (N= 65,047)**	
**Variable**	**Participants No. (%)**
**N (%)**	65,047
**Age, mean (SD)**	57.30 (7.77)
**Male, N, %**	26,982 (41.5)
**BMI, mean (SD)**	26.83 (4.60)
**Diabetes, N, %**	3,919 (6.0)
**Smoking history, N, %**	28,621 (44.0)
**HDL, mean (SD)**	56.49 (14.72)
**SBP, mean (SD)**	141.38 (19.69)
**Hypertension meds, N, %**	15373 (23.6)
**Total Cholesterol, mean (SD)**	222.06 (43.85)
**Statins, N, %**	11517 (17.7)
**eGFR, mean (SD)**	94.20 (12.79)
**BMI, CV, SD**	0.06 (0.04)
**N of BMI measurements, median, IQR**	4 (3-6)
**Stroke, N, %**	1,767 (2.7)
**MI, N, %**	5,136 (7.9)
**CV Death, N, %**	1,405 (2.2)
**MACE, N, %**	6,934 (10.7)

Abbreviations: BMI, body mass index; CAD, coronary artery disease; IQR, interquartile range; MACE, major adverse cardiac events; SD, standard deviation; CV, coefficient of variation; SBP, systolic blood pressure; HDL, high density lipoprotein.

© 2024 Almuwaqqat Z et al. *JAMA Network Open.*

Supplemental eTable 2. Unadjusted associations between body mass index (BMI) variability and adverse cardiovascular events.

Harmonized Ancestry and Race/Ethnicity	HR (95%CI) *	P
**NHW**	0.99 (0.94-1.04)	0.69
**NHB**	1.08 (1.00-1.16)	0.04
**HIS**	1.12 (0.98-1.28)	0.10

Abbreviation: HIS, Hispanic; HR, hazard ratio; NHB, Non-Hispanic Black; NHW, non-Hispanic White; 95%CI, 95% confidence interval *Per 1 standard deviation of BMI variability

Supplemental eTable 3. Associations Between Body Mass index (BMI) Variability and Adverse Cardiovascular Events from the UK Biobank

Outcome	HR (95%CI) *	P
Model 1		
**Incident CAD**	1.02 (0.99-1.04)	0.20
**Acute Ischemic Stroke**	1.01 (0.97-1.06)	0.53
**Cardiovascular Death**	1.08 (1.04-1.11)	<0.001
**MACE**	1.02 (0.995-1.04)	0.13
Model 2		
**Incident CAD**	1.02 (0.99-1.04)	0.20
**Acute Ischemic Stroke**	1.01 (0.97-1.06)	0.95
**Cardiovascular Death**	1.08 (1.04-1.11)	<0.001
**MACE**	1.02 (0.995-1.04)	0.12
Model 3		
**Incident CAD**	1.02 (0.99-1.05)	0.15
**Acute Ischemic Stroke**	1.00 (0.96-1.06)	0.87
**Cardiovascular Death**	1.08 (1.04-1.13)	<0.001
**MACE**	1.02 (0.99-1.04)	0.14

Abbreviation: HR, hazard ratio; 95%CI, 95% confidence interval; CAD, coronary artery disease; MACE, major adverse cardiac events.

*Per 1 standard deviation of BMI variability

Model 1 adjusted for age, sex, mean BMI, diabetes, smoking history, high-density lipoprotein, hypertension, total cholesterol level and estimated glomerular filtration rate.

Model 2= model 1 adjusted for BMI PRS

Model 3= model 1 adjusted for physical activity

© 2024 Almuwaqqat Z et al. *JAMA Network Open.*

Supplemental eTable 4. Sex-Specific Associations Between Body Mass index (BMI) Variability and Adverse Cardiovascular Events from the UK Biobank

Outcome	HR (95%CI) *	P
Male		
**Incident CAD**	1.01 (0.99-1.04)	0.43
**Acute Ischemic Stroke**	1.04 (0.99-1.10)	0.12
**Cardiovascular Death**	1.07 (1.02-1.12)	0.002
**MACE**	1.02 (0.99-1.05)	0.14
Female		
**Incident CAD**	1.03 (0.99-1.07)	0.19
**Acute Ischemic Stroke**	0.99 (0.92-1.06)	0.69
**Cardiovascular Death**	1.10 (1.03-1.11)	0.002
**MACE**	1.02 (0.98-1.05)	0.41

Abbreviation: HR, hazard ratio; 95%CI, 95% confidence interval; CAD, coronary artery disease; MACE, major adverse cardiac events.

*Per 1 standard deviation of BMI variability

Model adjusted for age, sex, mean BMI, diabetes, smoking history, high-density lipoprotein, hypertension, total cholesterol level and estimated glomerular filtration rate.

© 2024 Almuwaqqat Z et al. *JAMA Network Open.*

eAppendix. VA Million Veteran Program: Core Acknowledgment for Publications, February 2023. **MVP Program Office:** Sumitra Muralidhar, Ph.D., Program Director, US Department of Veterans Affairs, 810 Vermont Avenue NW, Washington, DC 20420; Jennifer Moser, Ph.D., Associate Director, Scientific Programs US Department of Veterans Affairs, 810 Vermont Avenue NW, Washington, DC 20420; Jennifer E. Deen, B.S., Associate Director, Cohort & Public Relations US Department of Veterans Affairs, 810 Vermont Avenue NW, Washington, DC 20420. **MVP Executive Committee:** Co-Chair: Philip S. Tsao, Ph.D, VA Palo Alto Health Care System, 3801 Miranda Avenue, Palo Alto, CA 94304; Co-Chair: Sumitra Muralidhar, Ph.D., US Department of Veterans Affairs, 810 Vermont Avenue NW, Washington, DC 20420; J. Michael Gaziano, MD, M.P.H., VA Boston Healthcare System, 150 S. Huntington Avenue, Boston, MA 02130; Elizabeth Hauser, Ph.D., Durham VA Medical Center, 508 Fulton Street, Durham, NC 27705; Amy Kilbourne, Ph.D., M.P.H., VA HSR&D, 2215 Fuller Road, Ann Arbor, MI 48105; Shiuh-Wen Luoh, MD, Ph.D., VA Portland Health Care System, 3710 SW US Veterans Hospital Rd, Portland, OR 97239; Michael Matheny, MD, M.S., M.P.H., VA Tennessee Valley Healthcare System, 1310 24th Ave. South, Nashville, TN 37212; Dave Oslin, MD, Philadelphia VA Medical Center, 3900 Woodland Avenue, Philadelphia, PA 19104. **MVP Co-Principal Investigators**: J. Michael Gaziano, MD, M.P.H., VA Boston Healthcare System, 150 S. Huntington Avenue, Boston, MA 02130; Philip S. Tsao, Ph.D., VA Palo Alto Health Care System, 3801 Miranda Avenue, Palo Alto, CA 94304. **MVP Core Operations**: Lori Churby, B.S., Director, MVP Regulatory Affairs, VA Palo Alto Health Care System, 3801 Miranda Avenue, Palo Alto, CA 94304; Stacey B. Whitbourne, Ph.D., Director, MVP Cohort Management VA Boston Healthcare System, 150 S. Huntington Avenue, Boston, MA 02130; Jessica V. Brewer, M.P.H., Director, MVP Recruitment & Enrollment VA Boston Healthcare System, 150 S. Huntington Avenue, Boston, MA 02130; Shahpoor (Alex) Shayan, M.S., Director, MVP Recruitment and Enrollment Informatics, VA Boston Healthcare System, 150 S. Huntington Avenue, Boston, MA 02130; Luis E. Selva, Ph.D., Executive Director, MVP Biorepositories VA Boston Healthcare System, 150 S. Huntington Avenue, Boston, MA 02130; Saiju Pyarajan Ph.D., Director, Data and Computational Sciences VA Boston Healthcare System, 150 S. Huntington Avenue, Boston, MA 02130; Kelly Cho, M.P.H, Ph.D., Director, MVP Phenomics Data Core VA Boston Healthcare System, 150 S. Huntington Avenue, Boston, MA 02130; Scott L. DuVall, Ph.D., Director, VA Informatics and Computing Infrastructure (VINCI), VA Salt Lake City Health Care System, 500 Foothill Drive, Salt Lake City, UT 84148; Mary T. Brophy MD, M.P.H., Director, VA Central Biorepository VA Boston Healthcare System, 150 S. Huntington Avenue, Boston, MA 02130. **MVP Coordinating Centers**: MVP Coordinating Center, Boston - J. Michael Gaziano, MD, M.P.H., VA Boston Healthcare System, 150 S. Huntington Avenue, Boston, MA 02130; MVP Coordinating Center, Palo Alto – Philip S. Tsao, Ph.D., VA Palo Alto Health Care System, 3801 Miranda Avenue, Palo Alto, CA 94304. **MVP Information Center**, Canandaigua – Brady Stephens, M.S., Canandaigua VA Medical Center, 400 Fort Hill Avenue, Canandaigua, NY 14424. Cooperative Studies Program Clinical Research Pharmacy Coordinating Center, Albuquerque – Todd Connor, Pharm.D.; Dean P. Argyres, B.S., M.S., New Mexico VA Health Care System, 1501 San Pedro Drive SE, Albuquerque, NM 87108. **MVP Publications and Presentations Committee**: Co-Chair: Tim Assimes, MD, VA Palo Alto Health Care System, 3801 Miranda Avenue, Palo Alto, CA 94304; Co-Chair: Adriana Hung, MD, VA Tennessee Valley Healthcare System, 1310 24th Ave. South, Nashville, TN 37212; Co-Chair: Henry Kranzler, MD, Philadelphia VA Medical Center, 3900 Woodland Avenue, Philadelphia, PA 19104. **MVP Local Site Investigators:** Samuel Aguayo, MD,

© 2024 Almuwaqqat Z et al. *JAMA Network Open.*

Phoenix VA Health Care System, 650 E. Indian School Road, Phoenix, AZ 85012; Sunil Ahuja, MD, South Texas Veterans Health Care System, 7400 Merton Minter Boulevard, San Antonio, TX 78229; Kathrina Alexander, MD, Veterans Health Care System of the Ozarks, 1100 North College Avenue, Fayetteville, AR 72703; Xiao M. Androulakis, MD, Columbia VA Health Care System, 6439 Garners Ferry Road, Columbia, SC 29209; Prakash Balasubramanian, MD, William S. Middleton Memorial Veterans Hospital, 2500 Overlook Terrace, Madison, WI 53705; Zuhair Ballas, MD, Iowa City VA Health Care System, 601 Highway 6 West, Iowa City, IA 52246-2208; Jean Beckham, Ph.D., Durham VA Medical Center, 508 Fulton Street, Durham, NC 27705; Sujata Bhushan, MD, VA North Texas Health Care System, 4500 S. Lancaster Road, Dallas, TX 75216; Edward Boyko, MD, VA Puget Sound Health Care System, 1660 S. Columbian Way, Seattle, WA 98108-1597; David Cohen, MD, Portland VA Medical Center, 3710 SW U.S. Veterans Hospital Road, Portland, OR 97239; Louis Dellitalia, MD, Birmingham VA Medical Center, 700 S. 19th Street, Birmingham AL 35233; L. Christine Faulk, MD, Robert J. Dole VA Medical Center, 5500 East Kellogg Drive, Wichita, KS 67218-1607; Joseph Fayad, MD, VA Southern Nevada Healthcare System, 6900 North Pecos Road, North Las Vegas, NV 89086; Daryl Fujii, Ph.D., VA Pacific Islands Health Care System, 459 Patterson Rd, Honolulu, HI 96819; Saib Gappy, MD, John D. Dingell VA Medical Center, 4646 John R Street, Detroit, MI 48201; Frank Gesek, Ph.D., White River Junction VA Medical Center, 163 Veterans Drive, White River Junction, VT 05009; Jennifer Greco, MD, Sioux Falls VA Health Care System, 2501 W 22nd Street, Sioux Falls, SD 57105; Michael Godschalk, MD, Richmond VA Medical Center, 1201 Broad Rock Blvd., Richmond, VA 23249; Todd W. Gress, MD, Ph.D., Hershel “Woody” Williams VA Medical Center, 1540 Spring Valley Drive, Huntington, WV 25704; Samir Gupta, MD, M.S.C.S., VA San Diego Healthcare System, 3350 La Jolla Village Drive, San Diego, CA 92161; Salvador Gutierrez, MD, Edward Hines, Jr. VA Medical Center, 5000 South 5th Avenue, Hines, IL 60141; John Harley, MD, Ph.D., Cincinnati VA Medical Center, 3200 Vine Street, Cincinnati, OH 45220; Kimberly Hammer, Ph.D., Fargo VA Health Care System, 2101 N. Elm, Fargo, ND 58102; Mark Hamner, MD, Ralph H. Johnson VA Medical Center 109 Bee Street, Mental Health Research, Charleston, SC 29401; Adriana Hung, MD, M.P.H., VA Tennessee Valley Healthcare System, 1310 24th Avenue, South Nashville, TN 37212; Robin Hurley, MD, W.G. (Bill) Hefner VA Medical Center, 1601 Brenner Ave, Salisbury, NC 28144; Pran Iruvanti, D.O., Ph.D., Hampton VA Medical Center, 100 Emancipation Drive, Hampton, VA 23667; Frank Jacono, MD, VA Northeast Ohio Healthcare System, 10701 East Boulevard, Cleveland, OH 44106; Darshana Jhala, MD, Philadelphia VA Medical Center, 3900 Woodland Avenue, Philadelphia, PA 19104; Scott Kinlay, M.B.B.S., Ph.D., VA Boston Healthcare System, 150 S. Huntington Avenue, Boston, MA 02130; Jon Klein, MD, Ph.D., Louisville VA Medical Center, 800 Zorn Avenue, Louisville, KY 40206; Michael Landry, Ph.D., Southeast Louisiana Veterans Health Care System, 2400 Canal Street, New Orleans, LA 70119; Peter Liang, MD, M.P.H., VA New York Harbor Healthcare System, 423 East 23rd Street, New York, NY 10010; Suthat Liangpunsakul, MD, M.P.H., Richard Roudebush VA Medical Center, 1481 West 10th Street, Indianapolis, IN 46202; Jack Lichy, MD, Ph.D., Washington DC VA Medical Center, 50 Irving St, Washington, D. C. 20422; C. Scott Mahan, MD, Charles George VA Medical Center, 1100 Tunnel Road, Asheville, NC 28805; Ronnie Marrache, MD, VA Maine Healthcare System, 1 VA Center, Augusta, ME 04330; Stephen Mastorides, MD, James A. Haley Veterans’ Hospital, 13000 Bruce B. Downs Blvd, Tampa, FL 33612; Elisabeth Mates MD, Ph.D., VA Sierra Nevada Health Care System, 975 Kirman Avenue, Reno, NV 89502; Kristin Mattocks, Ph.D., M.P.H., Central Western

© 2024 Almuwaqqat Z et al. *JAMA Network Open.*

Massachusetts Healthcare System, 421 North Main Street, Leeds, MA 01053; Paul Meyer, MD, Ph.D., Southern Arizona VA Health Care System, 3601 S 6th Avenue, Tucson, AZ 85723; Jonathan Moorman, MD, Ph.D., James H. Quillen VA Medical Center Corner of Lamont & Veterans Way, Mountain Home, TN 37684; Timothy Morgan, MD, VA Long Beach Healthcare System, 5901 East 7th Street Long Beach, CA 90822; Maureen Murdoch, MD, M.P.H., Minneapolis VA Health Care System, One Veterans Drive, Minneapolis, MN 55417; James Norton, Ph.D., VA Health Care Upstate New York, 113 Holland Avenue, Albany, NY 12208; Olaoluwa Okusaga, MD, Michael E. DeBakey VA Medical Center, 2002 Holcombe Blvd, Houston, TX 77030; Kris Ann Oursler, MD, Salem VA Medical Center, 1970 Roanoke Blvd, Salem, VA 24153; Ana Palacio, MD, M.P.H., Miami VA Health Care System, 1201 NW 16th Street, 11 GRC, Miami FL 33125; Samuel Poon, MD, Manchester VA Medical Center, 718 Smyth Road, Manchester, NH 03104; Emily Potter, Pharm.D., VA Eastern Kansas Health Care System, 4101 S 4th Street Trafficway, Leavenworth, KS 66048; Michael Rauchman, MD, St. Louis VA Health Care System, 915 North Grand Blvd, St. Louis, MO 63106; Richard Servatius, Ph.D., Syracuse VA Medical Center, 800 Irving Avenue, Syracuse, NY 13210; Satish Sharma, MD, Providence VA Medical Center, 830 Chalkstone Avenue, Providence, RI 02908; River Smith, Ph.D., Eastern Oklahoma VA Health Care System, 1011 Honor Heights Drive, Muskogee, OK 74401; Peruvemba Sriram, MD, N. FL/S. GA Veterans Health System, 1601 SW Archer Road, Gainesville, FL 32608; Patrick Strollo, Jr., MD, VA Pittsburgh Health Care System, University Drive, Pittsburgh, PA 15240; Neeraj Tandon, MD, Overton Brooks VA Medical Center, 510 East Stoner Ave, Shreveport, LA 71101; Philip Tsao, Ph.D., VA Palo Alto Health Care System, 3801 Miranda Avenue, Palo Alto, CA 94304-1290; Gerardo Villareal, MD, New Mexico VA Health Care System, 1501 San Pedro Drive, SE. Albuquerque, NM 87108; Agnes Wallbom, MD, M.S., VA Greater Los Angeles Health Care System, 11301 Wilshire Blvd, Los Angeles, CA 90073; Jessica Walsh, MD, VA Salt Lake City Health Care System, 500 Foothill Drive, Salt Lake City, UT 84148; John Wells, Ph.D., Edith Nourse Rogers Memorial Veterans Hospital, 200 Springs Road, Bedford, MA 01730; Jeffrey Whittle, MD, M.P.H., Clement J. Zablocki VA Medical Center, 5000 West National Avenue, Milwaukee, WI 53295; Mary Whooley, MD, San Francisco VA Health Care System, 4150 Clement Street, San Francisco, CA 94121; Allison E. Williams, N.D., Ph.D., R.N, Bay Pines VA Healthcare System, 10 000 Bay Pines Blvd Bay Pines, FL 33744; Peter Wilson, MD, Atlanta VA Medical Center, 1670 Clairmont Road, Decatur, GA 30033; Junzhe Xu, MD, VA Western New York Healthcare System, 3495 Bailey Avenue, Buffalo, NY 14215-1199; Shing Shing Yeh, Ph.D., MD, Northport VA Medical Center, 79 Middleville Road, Northport, NY 11768.

© 2024 Almuwaqqat Z et al. *JAMA Network Open.*
