# Supplementary material for: Long-Term Body Mass Index Variability and Adverse Cardiovascular Outcomes
Source: JAMA Netw Open. 2024 Mar 21;7(3):e243062. doi: 10.1001/jamanetworkopen.2024.3062 (PMC10958234; doi:10.1001/jamanetworkopen.2024.3062)
Supplement: Supplement 2. — Data Sharing Statement [file jamanetwopen-e243062-s002.pdf]

Data Sharing Statement

Almuwaqqat. Long-Term Body Mass Index Variability and Adverse Cardiovascular Outcomes. *JAMA Netw Open*. Published March 21, 2024. doi:10.1001/jamanetworkopen.2024.3062

Data
Data available: No

Additional Information
Explanation for why data not available: Due to US Department of Veterans Affairs (VA) regulations and our ethics agreements, the analytic datasets used for this study are not permitted to leave the Million Veteran Program (MVP) research environment and VA firewall. This limitation is consistent with other MVP studies based on VA data. However, the MVP data are made available to researchers with an approved VA and MVP study protocol. The UK Biobank data is available through the UK Biobank Showcase for researchers with approved projects.
